# LncRNA KCNQ1OT1 affects cell proliferation, apoptosis and fibrosis through regulating miR-18b-5p/SORBS2 axis and NF-ĸB pathway in diabetic nephropathy

**DOI:** 10.1186/s13098-020-00585-5

**Published:** 2020-09-03

**Authors:** Ran Jie, Pengpeng Zhu, Jiao Zhong, Yan Zhang, Hongyan Wu

**Affiliations:** 1 Department of Endocrinology, First People’s Hospital of Jingzhou, Shashi District, No. 8 Hangkong Road, Jingzhou, 434000 Hubei China; 2 Department of Anesthesiology, First People’s Hospital of Jingzhou, Jingzhou, 434000 Hubei China; 3 Health Management Center, First People’s Hospital of Jingzhou, Jingzhou, 434000 Hubei China; 4 Department of Gastroenterology, First People’s Hospital of Jingzhou, Jingzhou, 434000 Hubei China

**Keywords:** KCNQ1OT1, Diabetic nephropathy, miR-18b-5p, SORBS2, NF-ĸB pathway

## Abstract

**Background:**

It has been reported that long non-coding RNAs (lncRNAs) play vital roles in diabetic nephropathy (DN). Our study aims to research the function of lncRNA KCNQ1OT1 in DN cells and the molecular mechanism.

**Methods:**

Human glomerular mesangial cells (HGMCs) and human renal glomerular endothelial cells (HRGECs) were cultured in high glucose (30 mM) condition as models of DN cells. KCNQ1 opposite strand/antisense transcript 1 (KCNQ1OT1) and miR-18b-5p levels were detected by quantitative real-time polymerase chain reaction (qRT-PCR). The mRNA and protein levels of Sorbin and SH3 domain-containing protein 2 (SORBS2), Type IV collagen (Col-4), fibronectin (FN), transcriptional regulatory factor-beta 1 (TGF-β1), Twist, NF-κB and STAT3 were measured by qRT-PCR and western blot. Cell viability was detected by cell counting kit-8 (CCK-8) assay for selecting the proper concentration of glucose treatment. Additionally, 3-(4,5-dimethyl-2-thiazolyl)-2,5-diphenyl-2-H-tetrazolium bromide (MTT) and flow cytometry assay were employed to determine cell proliferation and apoptosis, respectively. The targets of KCNQ1OT1 was predicted by online software and confirmed by dual-luciferase reporter assay.

**Results:**

KCNQ1OT1 and SORBS2 were elevated in DN. Both knockdown of KCNQ1OT1 and silencing of SORBS2 restrained proliferation and fibrosis and induced apoptosis in DN cells. Besides, Overexpression of SORBS2 restored the KCNQ1OT1 knockdown-mediate effects on proliferation, apoptosis and fibrosis in DN cells. In addition, miR-18b-5p served as a target of KCNQ1OT1 as well as targeted SORBS2. KCNQ1OT1 knockdown repressed NF-ĸB pathway.

**Conclusion:**

KCNQ1OT1 regulated DN cells proliferation, apoptosis and fibrosis via KCNQ1OT1/miR-18b-5p/SORBS2 axis and NF-ĸB pathway.

## Highlights:

1. KCNQ1OT1 and SORBS2 were upregulated in DN patients and cells.
2. Knockdown of either KCNQ1OT1 or SORBS2 repressed proliferation and fibrosis and facilitated apoptosis in DN cells.
3. Overexpression of SORBS2 restored the effects of KCNQ1OT1 knockdown on proliferation, fibrosis and apoptosis in DN cells.
4. KCNQ1OT1 regulated SORBS2 expression via targeting miR-18b-5p.
5. Knockdown of KCNQ1OT1 inhibited NF-ĸB pathway.

## Background

Diabetic nephropathy (DN), a serious complication of diabetes mellitus, is the most pervasive cause of end-stage renal disease worldwide [1]. The causes of DN are extremely complicated, including smoking, poor glycemic control, dyslipidemia, environmental and genetic factors. Renal fibrosis is one of the main pathological features of DN. Inflammation could modulate the transforming growth factor β (TGF-β) activities, which is the vital regulator of fibrosis [2]. TGF-β regulated renal fibrosis in DN by activating various molecules such as collagen and fibronectin (FN) [3]. Although many efforts have been done in the prevention and therapy of DN, morbidity and mortality are still very high. Therefore, it is very important to understand the theoretical basis of its pathogenesis and search for available therapeutic strategies.

Long noncoding RNAs (lncRNAs) are a kind of transcripts with more than 200 nucleotides and can’t code protein [4]. With the development of high-throughput sequencing technology, more and more lncRNAs have been identified. Growing evidence indicated that lncRNAs were related to various diseases, including DN [5, 6]. LncRNAs have been revealed to regulate the pathologic process of DN. For instance, lncRNA NONHSAG053901 contributes to proliferation, fibrosis and inflammation in DN [7]. Knockdown of lncRNA XIST suppresses renal interstitial fibrosis in DN [8]. Inversely, lncRNA TUG1 plays an inhibiting effect on the proliferation and fibrosis in DN [9]. Also, lncRNAs can serve as drug targets for the early therapy of DN [10]. LncRNA KCNQ1 opposite strand/antisense transcript 1 (KCNQ1OT1) has been reported to involve in diabetic cardiomyopathy development [11]. However, the effect and molecular mechanism of KCNQ1OT1 on regulating DN development still remain unknown.

MicroRNAs (miRNAs) are crucial regulators on gene expression, which can degrade mRNA or inhibit transcription of mRNA by combining with the 3′-untranslated region (UTR) region of target genes [12, 13]. A number of emerging research have indicated that miRNAs play vital roles in DN. MiR-382 was upregulated in DN, and silencing of miR-382 represses the proliferation of glomerular mesangial cells [14]. MiR-146a suppresses reactive oxygen species generation and inflammation, thereby playing a preventive effect in DN [15]. Besides, certain miRNAs have been pointed to have clinical significance, which are considered as potential biomarkers for the diagnosis of early DN [16].

Sorbin and SH3 domain-containing protein 2 (SORBS2), also known as ArgBP2, regulates cell adhesion and motility by modulating signaling downstream of growth factor receptors [17]. Previous studies reported that SORBS2 could interact with multiple actin regulators and affect cell migration [18–20]. In addition, SORBS2 has been indicated to involve in tumor progression in many cancers [21, 22]. Nakatani et al. revealed that SORBS2 was associated with DN [23]. However, the regulatory mechanism of SORBS2 in DN remains unclear.

In this study, the expression of KCNQ1OT1 and SORBS2 in DN patients and simulative DN cells were detected. The effects of KCNQ1OT1 and SORBS2 in simulative DN cells were explored by KCNQ1OT1 knockdown and SORBS2 silencing. Additionally, the regulatory mechanism between KCNQ1OT1 and SORBS2 was further investigated.

## Materials and methods

### Serum samples

Blood samples were collected from 30 patients with DN (DN group) and 30 healthy volunteers (normal group). DN patients were diagnosed in First People’s Hospital of Jingzhou, Hubei Province. Serum samples were separated from blood and stored at -80℃. All patients signed informed consents. This study had acquired approval from the Ethics Committee of First People’s Hospital of Jingzhou, Hubei Province.

### Cell culture

Human glomerular mesangial cells (HGMCs) and human renal glomerular endothelial cells (HRGECs) were obtained from ScienCell Research Laboratories (Carlsbad, CA, USA). 293 T cells were bought from BeNa Culture Collection (Beijing, China). Cells were cultured in Dulbecco’s modified Eagle medium (DMEM; Invitrogen, Carlsbad, CA, USA) with 10% fetal bovine serum (Invitrogen) at 37℃ with 5% CO_2_. HGMCs and HRGECs were stimulated with 5.5 mM glucose (normal group) or 30 mM glucose (high glucose group). High glucose-induced HGMCs and HRGECs were served as models of DN cells.

### Quantitative real-time polymerase chain reaction (qRT-PCR)

Total RNAs were isolated using TRIzol Reagent (Invitrogen) under the instructions. Subsequently, reverse-transcription was performed by PrimeScript RT Reagent Kit (TaKaRa, Dalian, China) and microRNA First-Strand cDNA Synthesis Kit (Sangon Biotech, China). SYBR Green Master Mix (TaKaRa) was used for gene amplification. Gene expressions were normalized by glyceraldehyde-3-phosphate dehydrogenase (GAPDH) and U6 and calculated by 2^−∆∆Ct^ method. The primers were listed in Table 1.

**Table. 1:** The primer sequences used for qRT-PCR

Gene	Forword primers (5′–3′)	Reverse primers (5′–3′)
KCNQ1OT1 SORBS2 miR-18b-5p TGF-β1 Twist NF-κB STAT3 GAPDH U6	CCTCCCTCACTGAGCTTTGG GATAAATGAATAATTCTCTTTGATG TGTGCAAATCCATGCAAAACTGA TACCATGCCAACTTCTGTCTGGGA GGC​CAGGTA​CAT​CGA​CTT​ CGTAAAAGGACATATGAGAC CAGCAGCTTGACACACGGTA GCTGCTGAGTATGTCGTGGAGT GCTTCGGCAGCACATATACTAAAA	GTGCGGACCCTATACGGAAG AATTACCTGGAAGCCAGGTATGAA GTGCAGGGTCCGAGGT ATGTTGGACAACTGCTCCACCTTG TCC​AGA​CCG​AGA​AGG​CGT​AG TGGTGGGAAACTCATCATAG AAACACCAAAGTGGCATGTGA AGTCTTCTGGGTGGCAGTGAT CGCTTCACGAATTTGCGTGTCAT

### Western Blot Analysis

RIPA solution (Thermo Fisher Scientific, Waltham, MA, USA) was applied to extract total proteins. Then equal amounts of proteins were subjected to 10% sodium dodecyl sulfate–polyacrylamide gel electrophoresis (SDS-PAGE) and shifted onto a polyvinylidene difluoride (PVDF) membrane (Millipore, Billerica, MA, USA). After blocked with 5% nonfat milk, the membrane was incubated with the primary antibodies overnight at 4℃. Next, the secondary antibody was used to incubate the membrane for 1.5 h at room temperature. The protein bands were showed by enhanced chemiluminescence reagents (Millipore). The primary antibodies against SORBS2, Col-4, FN, TGF-β1, Twist, NF-κB, STAT3 and GAPDH were bought from Abcam (Cambridge, United Kingdom).

### Cell viability

The viability of HGMCs was determined by cell counting kit-8 (CCK-8; Beyotime, Shanghai, China). Cells were placed into a 96-well plate with 0, 10, 20 or 30 mM glucose. After cultured for 48 h, the cells were added with CCK-8 reagent and incubated for 2 h. Finally, a microplate reader (Thermo Fisher Scientific) was used to measure the absorbance at 450 nm.

### Cell transfection

Small interfering RNA (siRNA) of KCNQ1OT1 and SORBS2 (si-KCNQ1OT1 and si-SORBS2), miR-18b-5p mimic (miR-18b-5p), miR-18b-5p inhibitor and their control fragments (si-NC and miR-NC) were acquired from GenePharma (Shanghai, China). The full-length sequences of KCNQ1OT1 and SORBS2 were respectively inserted into pcDNA3.1 (Invitrogen) for the overexpression of KCNQ1OT1 and SORBS2. High glucose-induced HGMCs and HRGECs were transfected with si-RNA (si-NC, si-KCNQ1OT1 or si-SORBS2), miRNA (miR-NC, miR-18b-5p or miR-18b-5p inhibitor) or vector (pcDNA, pcDNA-KCNQ1OT1 or pcDNA-SORBS2) by Lipofectamine 2000 reagent (Invitrogen).

### 3-(4,5-dimethyl-2-thiazolyl)-2,5-diphenyl-2-H-tetrazolium bromide (MTT)

Cell proliferation was tested by MTT assay. Transfected cells were cultured in a 96-well plate for 24 h and then added 20 µL of MTT (5 mg/mL). After incubation for 4 h, the supernatants were removed. Subsequently, the formazan crystals was dissolved with 150 µL of dimethyl sulfoxide. The proliferation was assessed via the absorbance at 490 nm wavelength using a microplate reader (Thermo Fisher Scientific).

### Flow cytometry assay

Apoptosis was detected using annexin V-fluorescein isothiocyanate (V-FITC)/ propidium iodide (PI) apoptosis kit (BD Bioscience, San Diego, CA, USA), cells transfected for 48 h were collected and added annexin V-FITC and PI. After incubation for 20 min in a dark condition, cell apoptosis was tested by flow cytometry (BD Bioscience).

### Dual-luciferase reporter assay

The putative binding sequences between KCNQ1OT1 and miR-18b-5p, miR-18b-5p and SORBS2 were predicted by starBase 3.0. The wild type sequences of KCNQ1OT1 (WT-KCNQ1OT1) and 3′-UTR of SORBS2 (WT-SORBS2 3′UTR) contain the predicted binding sites of miR-18b-5p, the mutant sequences of KCNQ1OT1 (MUT-KCNQ1OT1) and mutant 3′-UTR of SORBS2 (MUT-SORBS2 3′UTR) were cloned into pGL3 reporter vectors (Promega, Madison, WI, USA), respectively. The reporter vectors with miR-18b-5p or miR-NC were co-transfected into 293 T cells for 48 h. The activities of firefly and renilla luciferases were detected with a Dual-Luciferase reporter system (Promega).

### Statistical analysis

Each experiment was done with at least three biological replicates. Statistical analysis was conduct by SPSS 22.0. Data were expressed as mean ± standard deviation (SD). Student’s *t*-test or one-way analysis of variance (ANOVA) with Tukey test was used to compare the differences of two groups or multiple groups. *P*-value < 0.05 was considered statistically significant.

## Results

### KCNQ1OT1 and SORBS2 were upregulated in DN

**Fig. 1:**
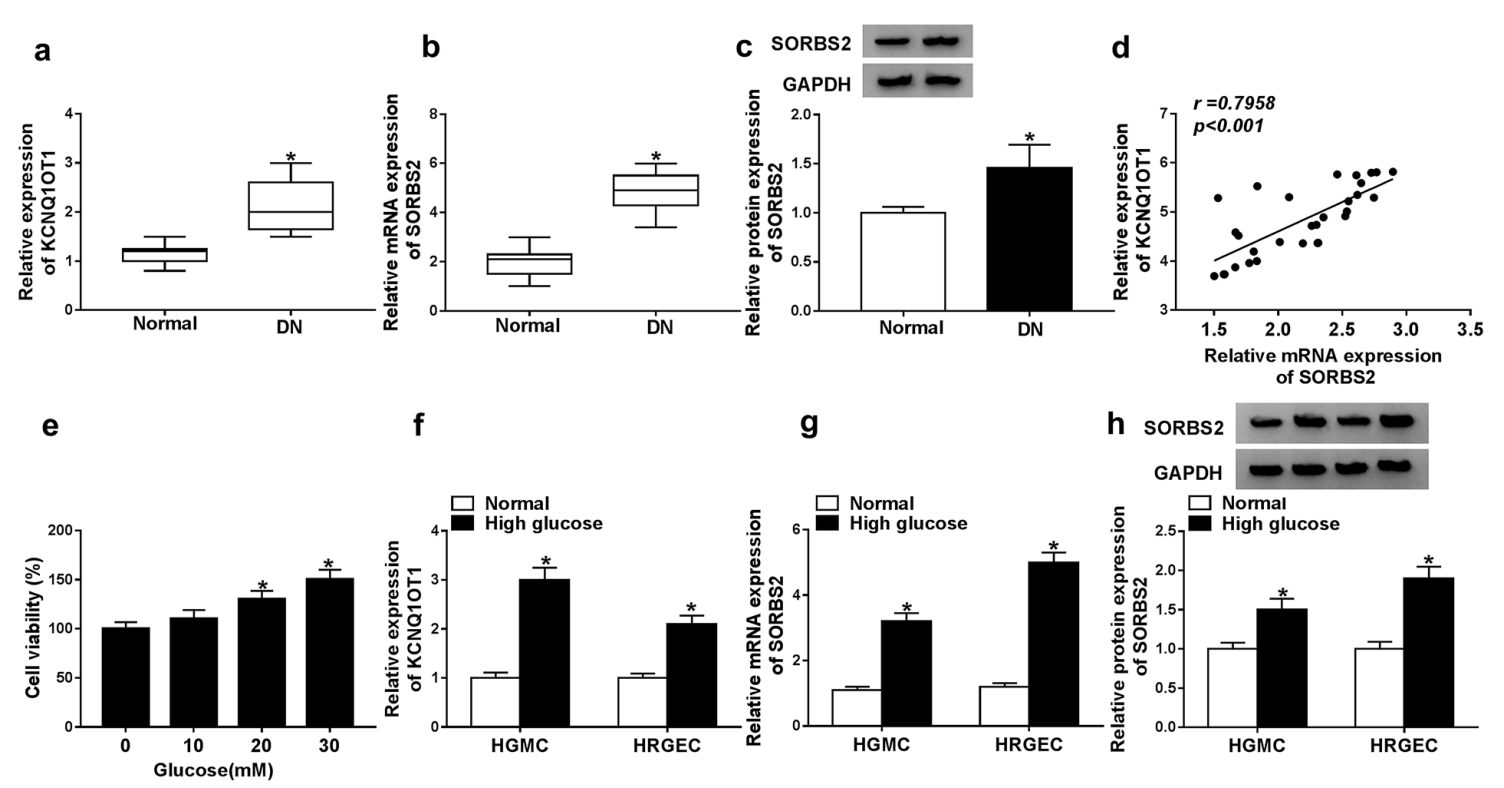
KCNQ1OT1 and SORBS2 were upregulated in DN patients and high glucose-induced HGMCs and HRGECs. **a** the expression of KCNQ1OT1 was detected in the serum of DN patients (n = 30) by qRT-PCR. **b**, **c** The mRNA and protein levels of SORBS2 were detected in the serum of DN patients (n = 30) by qRT-PCR and western blot. **d** the correlation of the KCNQ1OT1 level and SORBS2 mRNA level in the serum of DN patients was analyzed. **e** cell viability of HGMCs treated with 0, 10, 20, or 30 mM glucose was measured by CCK-8. **f** the expression of KCNQ1OT1 was detected in high glucose-induced and normal HGMCs and HRGECs by qRT-PCR. **g** and **h** the mRNA and protein levels were detected in high glucose-induced and normal HGMCs and HRGECs by qRT-PCR and western blot. N = 3, *P < 0.05

Firstly, KCNQ1OT1 level in the serum of DN patients was detected by qRT-PCR, and the result showed that the level of KCNQ1OT1 was significantly increased in DN patients compared with that in normal group (Fig. 1a). Next, the level of SORBS2 was measured by qRT-PCR and western blot, and the data revealed that SORBS2 was upregulated in DN patients (Fig. 1b, c). Besides, the levels of KCNQ1OT1 and SORBS2 mRNA had obviously positive correlation (Fig. 1d). Subsequently, we measured the viability of HGMCs treated with glucose. The cell viability was gradually enhanced with the increasing of glucose concentration (Fig. 1e). So 30 mM glucose was used for high glucose treatment to simulate the condition of DN. Additionally, KCNQ1OT1 and SORBS2 levels in HGMCs and HRGECs were detected. The results indicated that KCNQ1OT1 and SORBS2 were both elevated in high glucose group compared with normal group in both HGMCs and HRGECs (Fig. 1f, h). In addition, the cell viability was markedly increased and cell apoptosis rate was remarkably decreased in high glucose group compared with normal group (Additional file 1: Fig. S1). These data implied that KCNQ1OT1 and SORBS2 played vital roles in DN.

### Knockdown of KCNQ1OT1 repressed proliferation and induced apoptosis in DN cells.

**Fig. 2:**
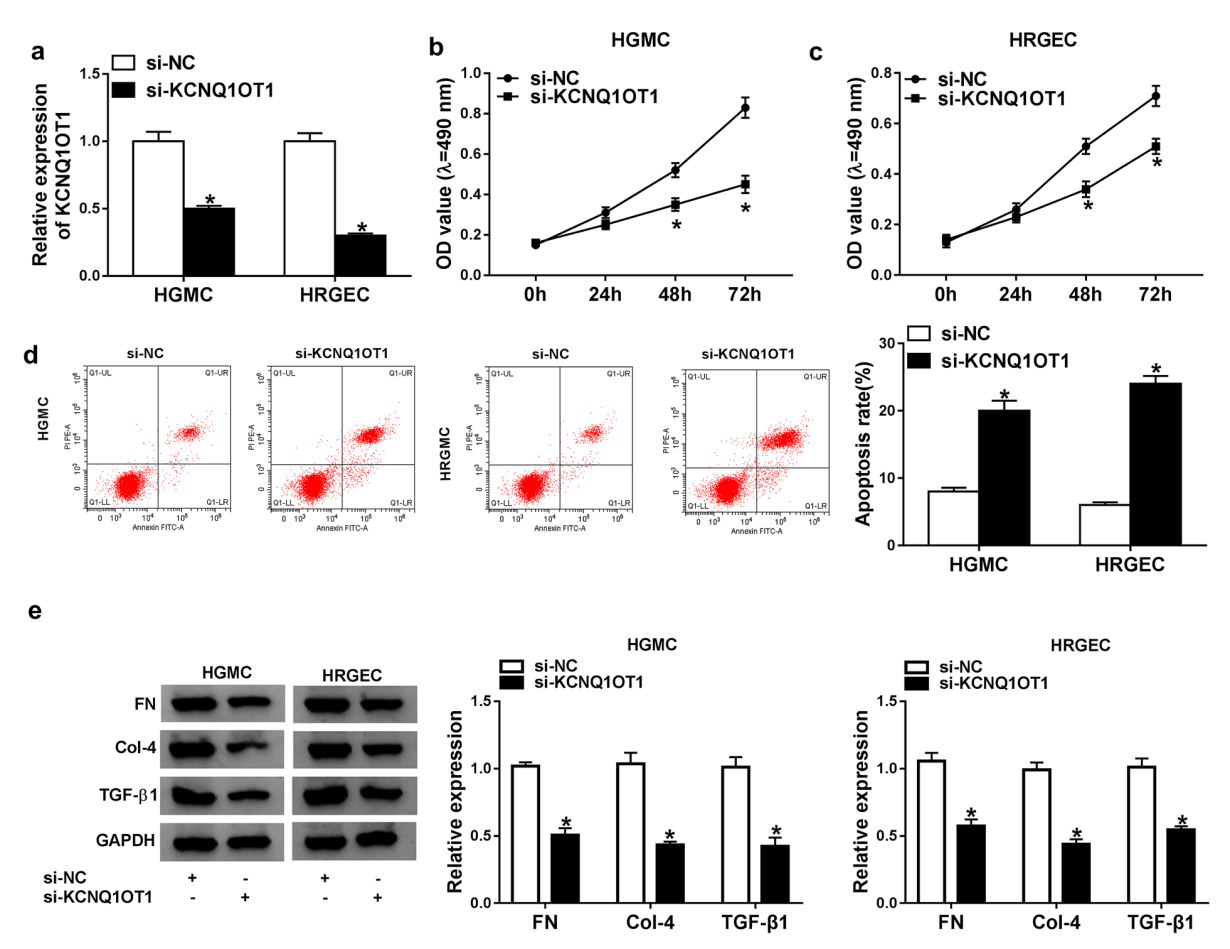
Knockdown of KCNQ1OT1 inhibited proliferation and fibrosis and promoted apoptosis in high glucose-induced HGMCs and HRGECs. High glucose-induced HGMCs and HRGECs were transfected with si-KCNQ1OT1. **a** The expression of KCNQ1OT1 was detected by qRT-PCR. **b**, **c** proliferation of HGMCs and HRGECs was determined by MTT assay. **d** apoptosis of HGMCs and HRGECs was determined by flow cytometry assay. Apoptotic rate: Annexin positive cells/all cells. **e** The expression of fibrosis-related proteins was detected by western blot assay in glucose-induced HGMCs and HRGECs. N = 3, *P < 0.05

To investigate the role of KCNQ1OT1 in DN cells, HGMCs and HRGECs were treated with high glucose and transfected with si-KCNQ1OT1. The qRT-PCR result revealed that KCNQ1OT1 level was obviously reduced in HGMCs and HRGECs transfected with si-KCNQ1OT1 (Fig. 2a). Subsequently, proliferation and apoptosis of HGMCs and HRGECs was assessed by MTT and Flow cytometry assay. MTT assay showed that KCNQ1OT1 knockdown suppressed cell proliferation in both HGMCs and HRGECs (Fig. 2b, c). On the contrary, apoptosis was promoted in HGMCs and HRGECs transfected with si-KCNQ1OT1 (Fig. 2d). In addition, knock-down of KCNQ1OT1 down-regulated the expression levels of FN, Col-4 and TGF-β1 in HFMC and HRGEC cells (Fig. 2e). These results proved that KCNQ1OT1 played a promoting effect on DN cells.

### Silencing of SORBS2 restrained proliferation and contributed to apoptosis in DN cells.

**Fig. 3:**
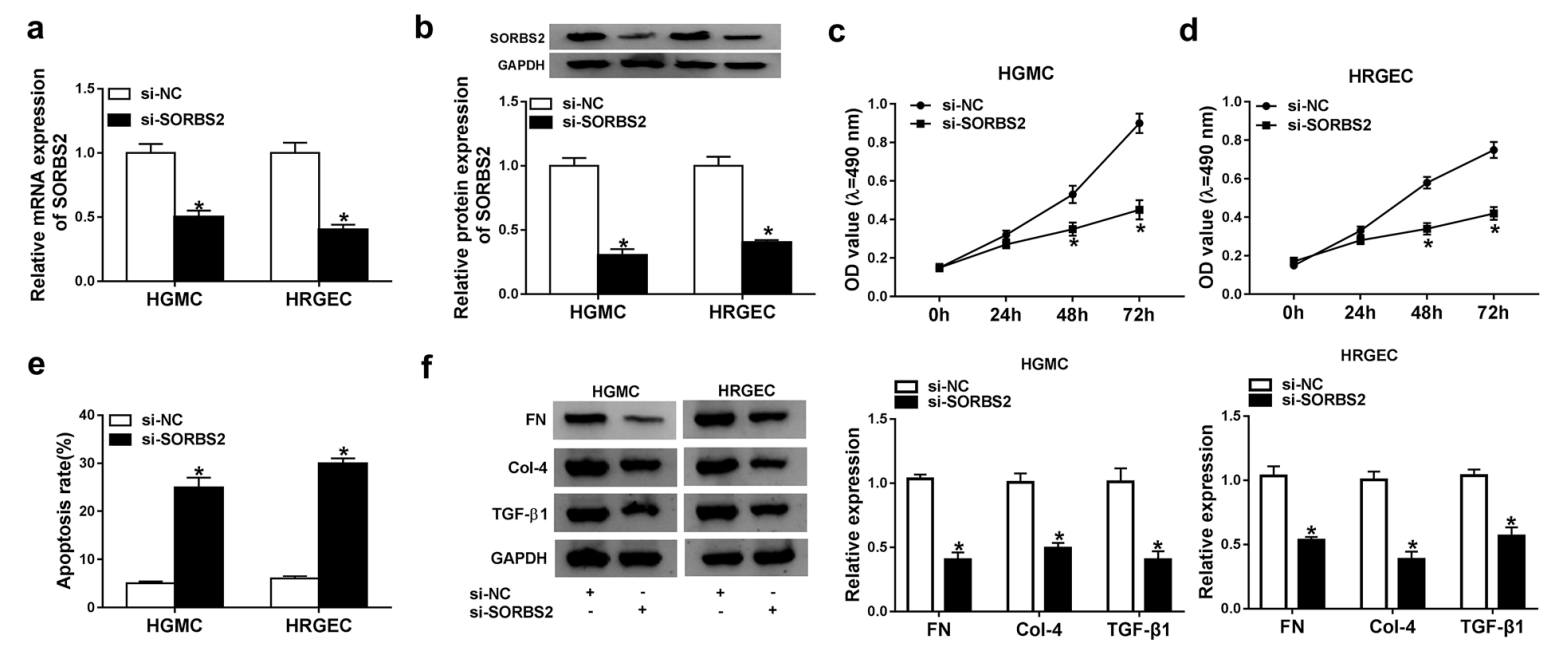
Silencing of SORBS2 repressed proliferation and facilitated apoptosis in high glucose-induced HGMCs and HRGECs. High glucose-induced HGMCs and HRGECs were transfected with si-SORBS2. **a**, **b** The mRNA and protein levels of SORBS2 were detected by qRT-PCR and western blot. **c**, **d** proliferation of HGMCs and HRGECs was determined by MTT assay. **e** apoptosis of HGMCs and HRGECs was determined by flow cytometry assay. **f** The expression of fibrosis-related proteins was measured by western blot assay. N = 3, *P < 0.05

To explore the effects of SORBS2 in DN in vitro, high glucose-induced HGMCs and HRGECs were transfected with si-SORBS2. Then the expression of SORBS2 was measured to evaluate the silencing efficiency. The mRNA level of SORBS2 was significantly downregulated in HGMCs and HRGECs transfected with si-SORBS2 (Fig. 3a). And the protein level of SORBS2 in HGMCs and HRGECs consisted with the mRNA level (Fig. 3b). Subsequently, MTT assay implied that proliferation was inhibited in HGMCs and HRGECs transfected with si-SORBS2 (Fig. 3c, d). In addition, silencing of SORBS2 facilitated apoptosis of HGMCs and HRGECs (Fig. 3e). Furthermore, the fibrosis related proteins FN, Col-4 and TGF-β1 were significantly decreased after transfection with si-SORBS2 (Fig. 3f). These data indicated that downregulation of SORBS2 suppressed proliferation and fibrosis and promoted apoptosis in DN in vitro.

### KCNQ1OT1 impacted proliferation, fibrosis and apoptosis by regulating SORBS2 expression in DN cells.

**Fig. 4:**
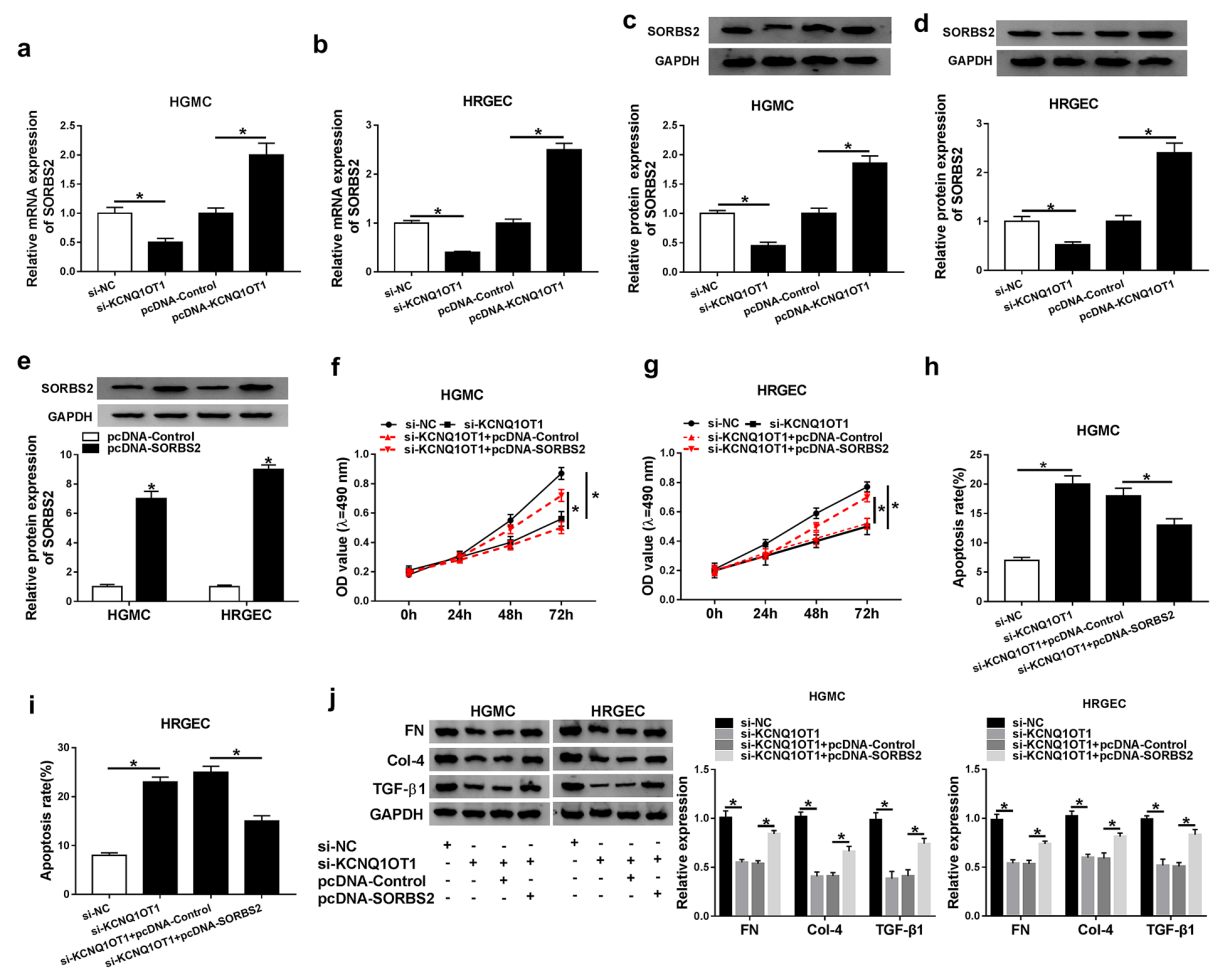
KCNQ1OT1 affected proliferation and apoptosis by regulating SORBS2 expression in high glucose-induced HGMCs and HRGECs. **a**, **b** the mRNA level of SORBS2 was detected by qRT-PCR in HGMCs and HRGECs transfected with si-KCNQ1OT1 or pcDNA-KCNQ1OT1. **c**, **d** the protein level of SORBS2 was detected by western blot in HGMCs and HRGECs transfected with si-KCNQ1OT1 or pcDNA-KCNQ1OT1. **e** the protein level of SORBS2 was detected in HGMCs and HRGECs transfected with pcDNA-SORBS2. **f**, **g**, proliferation of HGMCs and HRGECs was determined after transfected with si-KCNQ1OT1 or si-KCNQ1OT1 + pcDNA-SORBS2. **h** and **i** apoptosis of HGMCs and HRGECs was determined after transfected with si-KCNQ1OT1 or si-KCNQ1OT1 + pcDNA-SORBS2. **j** western blot assay was utilized to detect the fibrosis-related proteins. N = 3, *P < 0.05

To investigate the relationship between KCNQ1OT1 and SORBS2 in DN cells, the mRNA and protein levels of SORBS2 were detected in high glucose-induced HGMCs and HRGECs after KCNQ1OT1 was silenced or overexpressed. The qRT-PCR result suggested the mRNA level of SORBS2 was downregulated by knockdown of KCNQ1OT1 while increased by overexpression of KCNQ1OT1 in HGMCs and HRGECs (Fig. 4a, b). Similarly, the protein level of SORBS2 was reduced by si-KCNQ1OT1 but elevated by pcDNA-KCNQ1OT1 in HGMCs and HRGECs (Fig. 4c, d). Additionally, pcDNA-SORBS2 was used to overexpress SORBS2, and the western blot result showed that the protein level of SORBS2 was increased in HGMCs and HRGECs transfected with pcDNA-SORBS2 (Fig. 4e). Then, proliferation and apoptosis was determined in HGMCs and HRGECs transfected with si-KCNQ1OT1 or si-KCNQ1OT1 + pcDNA-SORBS2. And the results exhibited cell proliferation was impeded by KCNQ1OT1 knockdown and rescued by SORBS2 overexpression in HGMCs and HRGECs (Fig. 4f, g). Inversely, apoptosis was promoted in HGMCs and HRGECs transfected with si-KCNQ1OT1 and reversed when co-transfected with pcDNA-SORBS2 (Fig. 4h, i). In addition, the expression levels of fibrosis-related proteins FN, Col-4 and TGF-β1 were markedly down-regulated after transfection with si-KCNQ1OT1, while overexpression of SORBS2 reversed the changes of fibrosis-related proteins caused by KCNQ1OT1 knockdown (Fig. 4j). These results manifested that KCNQ1OT1 affected proliferation, apoptosis and fibrosis through regulating SORBS2 expression in DN in vitro.

### KCNQ1OT1 regulated SORBS2 expression via directly targeting miR-18b-5p

**Fig. 5:**
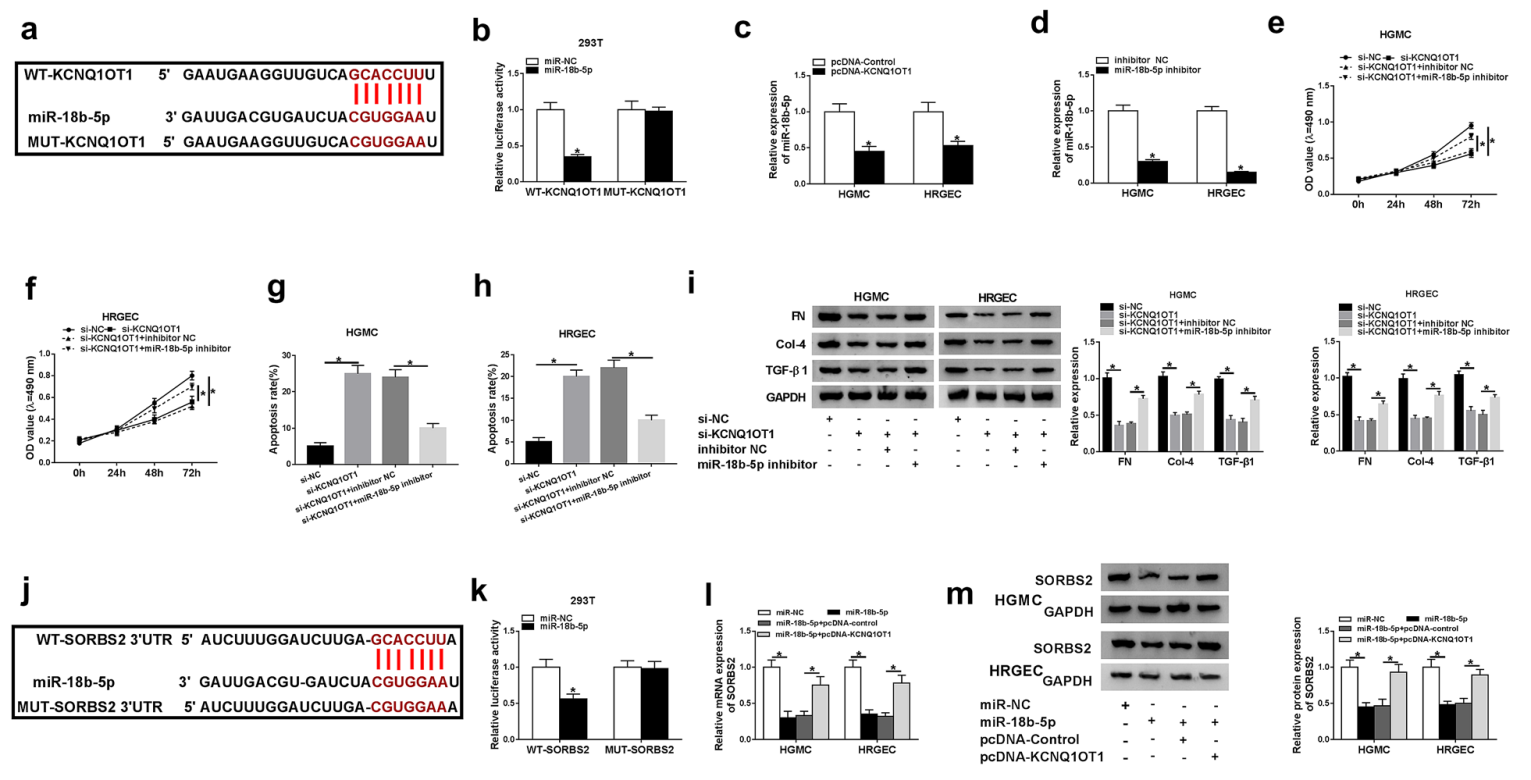
KCNQ1OT1 targeted miR-18b-5p to regulate SORBS2 expression. **a** the putative binding sites between KCNQ1OT1 and miR-18b-5p were predicated by starBase 3.0. **b** The luciferase activities of WT-KCNQ1OT1 and MUT-KCNQ1OT1 were measured in 293 T cells transfected with miR-NC or miR-18b-5p. **c** The expression of miR-18b-5p was detected by qRT-PCR in HGMCs and HRGECs transfected with pcDNA-KCNQ1OT1. **d** The expression of miR-18b-5p was detected by qRT-PCR in HGMCs and HRGECs transfected with miR-18b-5p inhibitor. **e**, **f** proliferation of HGMCs and HRGECs was determined after transfected with si-KCNQ1OT1 or si-KCNQ1OT1 + miR-18b-5p inhibitor. **g**, **h** apoptosis of HGMCs and HRGECs was determined after transfected with si-KCNQ1OT1 or si-KCNQ1OT1 + miR-18b-5p inhibitor. **i** the fibrosis-related proteins were detected by western blot after transfection with si-KCNQ1OT1 or si-KCNQ1OT1 + miR-18b-5p inhibitor. **j** The putative binding sites between miR-18b-5p and SORBS2 were predicated by starBase 3.0. **k** The luciferase activities of WT-SORBS2 and MUT- SORBS2 were measured in 293 T cells transfected with miR-NC or miR-18b-5p. **l**, **m**, the mRNA and protein levels of SORBS2 were detected in HGMCs and HRGECs transfected with miR-18b-5p or miR-18b-5p + pcDNA-KCNQ1OT1. N = 3, *P < 0.05

To further explore the regulatory mechanism between KCNQ1OT1 and SORBS2, the target of KCNQ1OT1 was predicated by starBase 3.0. MiR-18b-5p was predicated as a potential target and the putative binding sequences for KCNQ1OT1 were exhibited (Fig. 5a). WT-KCNQ1OT1 and MUT-KCNQ1OT1 was constructed for dual-luciferase reporter assay. The luciferase activity was remarkably decreased by miR-18b-5p in 293 T cells transfected with WT-KCNQ1OT1 and had no change in MUT-KCNQ1OT1 group (Fig. 5b). Moreover, the expression of miR-18b-5p was measured in HGMCs and HRGECs transfected with pcDNA-KCNQ1OT1, and the result indicated that overexpression of KCNQ1OT1 downregulated the expression of miR-18b-5p in HGMCs and HRGECs (Fig. 5c). Also, the expression of miR-18b-5p was greatly repressed by miR-18b-5p inhibitor (Fig. 5d). In order to investigate the interaction between KCNQ1OT1 and miR-18b-5p in DN cells, high glucose-induced HGMCs and HRGECs were transfected with si-KCNQ1OT1 or si-KCNQ1OT1 + miR-18b-5p inhibitor. Cell proliferation was inhibited by KCNQ1OT1 knockdown and reverted by miR-18b-5p inhibitor in HGMCs and HRGECs (Fig. 5e, f). Besides, the enhanced apoptosis of HGMCs and HRGECs by si-KCNQ1OT1 was abolished by inhibiting miR-18b-5p (Fig. 5g, h). Furthermore, the fibrosis-related proteins (FN, Col-4 and TGF-β1) were down-regulated by KCNQ1OT1 knockdown and reversed by transfection with miR-18b-5p inhibitor (Fig. 5i). Interestingly, starBase 3.0 showed that miR-18b-5p can bind to 3′UTR of SORBS2 (Fig. 5j). Dual-luciferase reporter assay presented that the luciferase activity was weakened by miR-18b-5p in 293 T cells transfected with WT-SORBS2 3′UTR rather than MUT-SORBS2 3′UTR (Fig. 5k). In addition, the mRNA and protein levels of SORBS2 were determined in high glucose-induced HGMCs and HRGECs transfected with miR-18b-5p or miR-18b-5p and pcDNA-KCNQ1OT1. The data displayed that the expression of SORBS2 was attenuated by miR-18b-5p but rescued by KCNQ1OT1 overexpression (Fig. 5l, m). These results suggested that KCNQ1OT1 served as a competing endogenous RNA (ceRNA) to upregulate SORBS2 expression by sponging miR-18b-5p, thereby affecting proliferation and apoptosis in DN cells.

### Knockdown of KCNQ1OT1 repressed NF-ĸB pathway

**Fig. 6:**
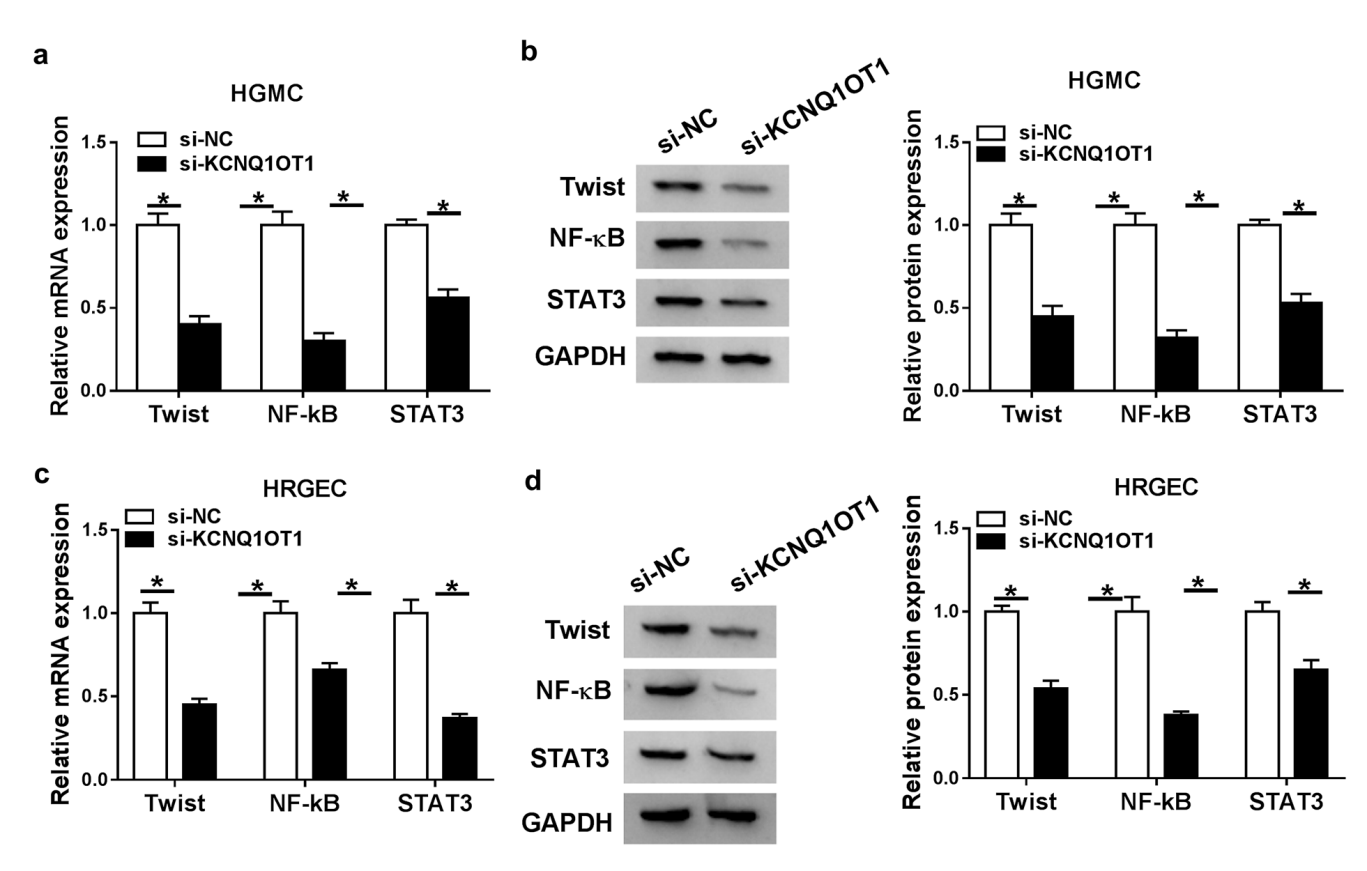
Knockdown of KCNQ1OT1 inhibited EMT and NF-ĸB pathway.** a**, **b** the mRNA and protein levels of Twist, NF-κB and STAT3 were detected in HGMCs transfected with si-KCNQ1OT1. **c**, **d** the mRNA and protein levels of Twist, NF-κB and STAT3 were detected in HRGECs transfected with si-KCNQ1OT1. N = 3, *P < 0.05

The expression levels NF-ĸB pathway-related proteins (Twist, NF-κB and STAT3) were detected to assess NF-ĸB pathway. High glucose-induced HGMCs and HRGECs were transfected with si-KCNQ1OT1 for the knockdown of KCNQ1OT1. The results showed the mRNA and protein levels of Twist, NF-κB and STAT3 were all decreased in HGMC cells transfected with si-KCNQ1OT1 (Fig. 6a, b), and there was a same trend in HRGEC cells (Fig. 6c, d), which suggesting that knockdown of KCNQ1OT1 impeded NF-ĸB pathway in DN cells.

## Discussion

Diabetes mellitus is a very prevalent disease, and its morbidity is increasing rapidly around the world. DN results from diabetic angiopathy, which is a great threat to the patient's life. Recent studies suggest that lncRNAs play vital roles in the development of DN. However, the precise mechanism remains unclear.

KCNQ1OT1 has been revealed to associate with various diseases. It was reported that KCNQ1OT1 was upregulated and promoted tumor development in osteosarcoma, non-small cell lung cancer, colorectal cancer and so on [24–26]. Moreover, KCNQ1OT1 restrained apoptosis and contributed to cell proliferation in acute myeloid leukemia [27]. On the contrary, KCNQ1OT1 facilitated autophagy in cerebral ischemic stroke [28]. In addition, KCNQ1OT1 was overexpressed in diabetic and induced pyroptosis in diabetic cardiomyopathy [11]. In this study, KCNQ1OT1 was upregulated in DN patients and high glucose-induced HGMCs and HRGECs. Cell proliferation and fibrosis were inhibited and apoptosis was induced by knockdown of KCNQ1OT1, indicating that KCNQ1OT1 plays a promoting effect in DN progression.

LncRNAs function as ceRNAs to bind to miRNAs and regulate the expression of miRNAs. Online software showed that miR-18b-5p contained putative binding sites of KCNQ1OT1, and dual-luciferase reporter assay confirmed that miR-18b-5p was a target of KCNQ1OT1. MiR-18b-5p was manifested to impede lung adenocarcinoma cells proliferation but promote breast cancer development [29, 30]. In addition, a previous study proved that miR-18b played an inhibitory role in the progression of DN [31]. Also, Wu et al. displayed that miR-18b repressed the proliferation of high glucose-induced human retinal endothelial cells. Consistent with these findings, our data demonstrated that silencing of miR-18b-5p reversed the inhibitory effects mediated by KCNQ1OT1 knockdown on high glucose-induced HGMCs and HRGECs.

SORBS2 was closely related to cell adhesion, and it localized in actin stress fibers [32]. Proteomics analysis presented that SORBS2 was obviously upregulated in the diabetic cardiomyopathy model [33]. And another study suggested SORBS2 involved in DN progression through regulating actin filaments [23]. Here, we found that SORBS2 expression was notably increased in DN patients and high glucose-induced HGMCs and HRGECs, and the mRNA level of SORBS2 was positively correlated with KCNQ1OT1 expression. Silencing of SORBS2 suppressed proliferation and fibrosis and enhanced apoptosis of HGMCs and HRGECs under high glucose conditions. Moreover, the effect of SORBS2 overexpression has an antagonism against KCNQ1OT1 knockdown in high glucose-induced HGMCs and HRGECs. Additionally, SORBS2 was identified as the downstream target of miR-18b-5p, and KCNQ1OT1 regulated SORBS2 expression through miR-18b-5p.

It is well known that NF-κB signaling pathway plays an important role in disease progression. A previous study manifested that STAT3/NF-κB pathway facilitated inflammation and ECM accumulation in high glucose-induced human mesangial cells [34]. NF-κB and STAT3 have been revealed to regulate inflammation and cell adhesion [35]. In addition, Twist had the function of regulating NF-κB pathway [36]. In our study, the expression of Twist, NF-κB and STAT3 were detected to evaluate the NF-κB signaling pathway in high glucose-induced HGMCs and HRGECs. The data showed that Twist, NF-κB and STAT3 were downregulated after transfection with si-KCNQ1OT1, which indicated KCNQ1OT1 knockdown inhibited DN development through the NF-κB pathway. However, the limitation of our study is loss of in vivo experiments and further studies were needed in animals to research the function of KCNQ1OT1 in DN in vivo.

## Conclusion

In conclusion, our study first demonstrated that KCNQ1OT1 was upregulated in DN and played a promoting role in the development of DN. Further mechanism research indicated that KCNQ1OT1 promoted proliferation and restrained apoptosis via miR-18b-5p/SORBS2 axis in high glucose-induced HGMCs and HRGECs. Moreover, KCNQ1OT1 knockdown inhibited EMT and NF-κB pathway. This study elucidated the role of KCNQ1OT1/miR-18b-5p/SORBS2 axis in DN, which might provide a new light for the diagnosis and treatment of DN.

## Supplementary information

**Additional file 1: Figure S1.** High glucose induced proliferation and inhibited apoptosis of renal cells. HGMCs and HRGECs were treated with high glucose (30 mM). **a** The proliferation of HGMCs and HRGECs was determined by MTT assay. **b** Apoptosis of HGMCs and HRGECs was determined by flow cytometry assay. N = 3, *P < 0.05.

## Data Availability

All data generated or analyzed during this study are included in this published Article.
