# Supplementary figures and images for: LncRNA KCNQ1OT1 affects cell proliferation, apoptosis and fibrosis through regulating miR-18b-5p/SORBS2 axis and NF-ĸB pathway in diabetic nephropathy
Source: Diabetol Metab Syndr. 2020 Sep 3;12:77. doi: 10.1186/s13098-020-00585-5 (PMC7469295; doi:10.1186/s13098-020-00585-5)

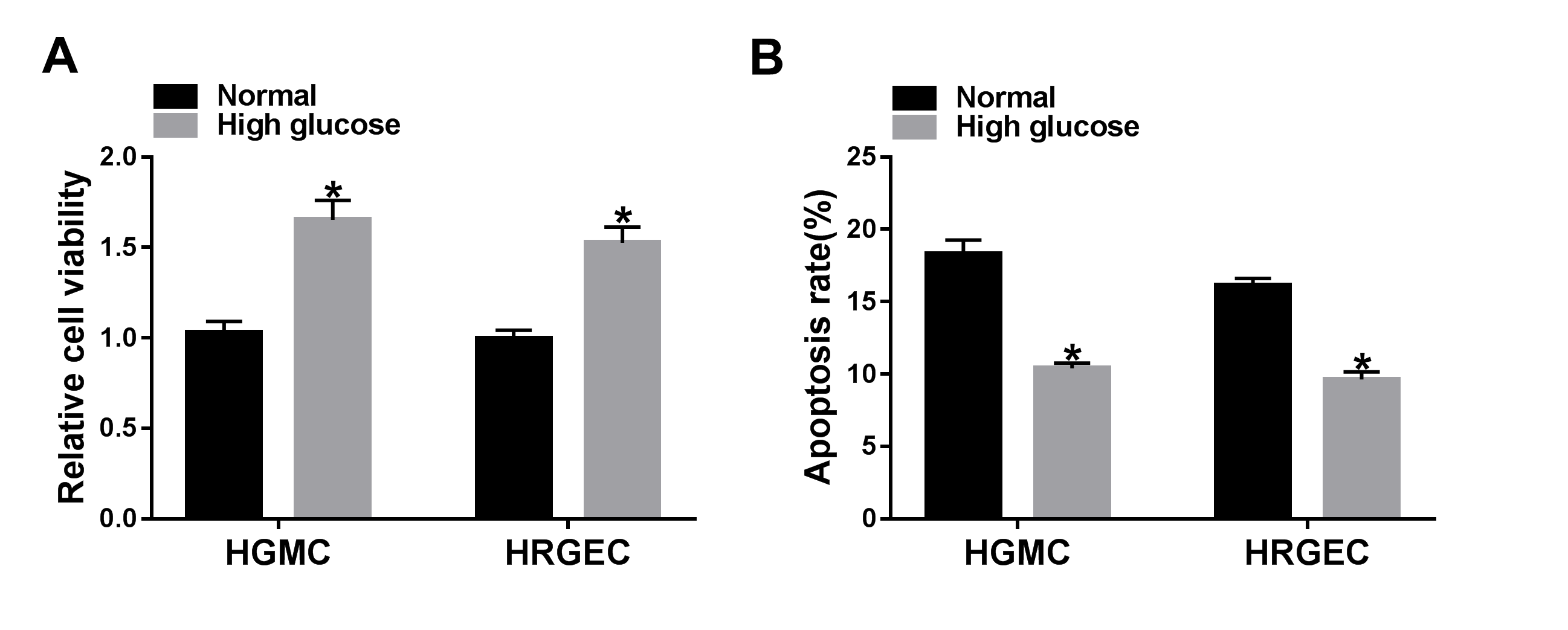

Supplement: Supplementary file 1 — Additional file 1: Figure S1. High glucose induced proliferation and inhibited apoptosis of renal cells. HGMCs and HRGECs were treated with high glucose (30 mM). a The proliferation of HGMCs and HRGECs was determined by MTT assay. b Apoptosis of HGMCs and HRGECs was determined by flow cytometry assay. N = 3, *P < 0.05. [file 13098_2020_585_MOESM1_ESM.tif]
